# Outpatient endoscopic resection of large calcified thoracic disc herniation with caudal displacement

**DOI:** 10.3171/2021.10.FOCVID2112

**Published:** 2022-01-01

**Authors:** Sanjay Konakondla, Nelson Sofoluke, Sean M. Barber, Sarah A. Rimini, Jonathan R. Slotkin

**Affiliations:** 1 Geisinger Neuroscience Institute, Geisinger, Danville, Pennsylvania;; 2 Department of Neurosurgery, Houston Methodist Neurological Institute, Houston, Texas; and; 3 Radiology 3D Lab, Geisinger, Danville, Pennsylvania

**Keywords:** endoscopic spine surgery, thoracic disc, ambulatory surgery

## Abstract

Thoracic disc herniations can cause radiculopathy and myelopathy from neural compression. Surgical resection may require complex, morbid approaches. To avoid spinal cord retraction, wide exposures requiring extensive tissue, muscle, and bony disruption are needed, which may require instrumentation. Anterior approaches may require vascular surgeons, chest tube placement, and intensive care admission. Large, calcified discs or migrated fragments can pose additional challenges. Previous literature has noted the endoscopic approach to be contraindicated for calcified thoracic discs. The authors describe an ultra–minimally invasive, ambulatory endoscopic approach to resect a large calcified thoracic disc with caudal migration and avoidance of conventional approaches.

The video can be found here: https://stream.cadmore.media/r10.3171/2021.10.FOCVID2112

## Transcript

### 0:35 Introduction and Indications.

The patient is a 32-year-old female, BMI 54, who was suffering from 6 months of midthoracic back pain, progressive gait abnormalities, heaviness, and weakness in the legs. At the time of surgery, she was using a walker to assist with ambulation.

### 0:50 Imaging.

Imaging included an MRI and subsequently a CT of the thoracic spine which showed a large, calcified disc at the level of T9–10, preferential to the right side with caudal migration and significant spinal cord compression. A surgery was felt necessary for this patient due to her significant clinical and radiographic findings.^1^

### 1:10 Digital 3D Rendering.

To appreciate the intraoperative view and anticipate the amount of bony resection necessary, 3D models were digitally reconstructed from the patient’s CT scans and MRI. Areas were subtracted to allow the best possible view of the disc and to plan trajectories for complete removal of the calcified disc and the caudally migrated fragment. 3D-printed models were fashioned to facilitate operative planning. We often use these 3D models for patient education and surgical discussion.

### 1:45 Patient Positioning.

Patient positioning is typical as with any discectomy, with the patient in a prone position on a Wilson frame to facilitate opening of the neural foramen. Marking levels intraoperatively, as always, is done carefully with specific reference to preoperative imaging when comparing to intraoperative fluoroscopic images. Due to the patient’s body habitus, the calcified disc was unable to be unambiguously seen, contrary to what we would have predicted. We counted up from the 12th rib but also from the sacrum to mark the level of T9–10.

### 2:20 Intraoperative Planning and Marking.

Key anatomical lines are drawn on the patient to mark the midline, the disc level, and trajectories. In a typical endoscopic transforaminal approach, the guide needle is docked on the posterior superior apex of the inferior vertebral body (on the lateral view) and just medial to the ipsilateral pedicle (on the AP view).^2^ Due to the large calcified caudal migration of this disc, it would have not been helpful to dock in this area, so we proceeded with a modified transforaminal approach. The ideal trajectory as determined by our 3D model suggested a more acute angle aimed inferiorly, which could better be categorized as a combined transforaminal and ventrolateral transpedicular approach. The needle as seen is docked just lateral to the pedicle at the pedicle–superior articulating process (SAP)–transverse process (TP) junction of T10. A < 1-cm incision was made around the needle, a guidewire was placed into the needle before the needle was removed, and sequential dilators were advanced over the guidewire to protect surrounding tissue. The working channel was advanced over the final dilator.

### 3:29 Tissue Exposure.

The endoscope was introduced. In this case, the tubular retractor had an inner diameter of 6.5 mm, facilitating the 6.3-mm outer diameter of the endoscope, which has a working channel diameter of 3.7 mm. The surrounding tissue was removed and cauterized to reveal the underlying bony anatomy. The inferior articulating process (IAP) of T9, the SAP, TP, and the proximal pedicle of T10 is visualized. An instrument can then be placed lateral to the pedicle of T10 on the superior border and an intraoperative fluoroscopic image can be taken.

### 4:05 Drilling of Bony Anatomy.

A high-speed 3.5-mm round diamond burr was used to begin the planned bony resection including partial SAP, TP, and pedicle of T10 and T9 IAP.

### 4:20 Joint Visualization.

The T9–10 joint space can be visualized here easily after minimal drilling. The pedicle is identified anatomically and confirmed again with fluoroscopic imaging. After trajectory of bony drilling was verified, drilling continued to the medial surface of the pedicle.

### 4:40 Pedicle Landmark.

The medial cortical surface of the T10 pedicle can be visualized here, labeled in the still image. Intraoperative fluoroscopy was taken to confirm these live anatomical landmarks. We continued drilling in the cephalad direction to remove the remaining T10 SAP. Bleeding from the epidural veins allows for appreciation of entrance into the epidural space. These veins are carefully cauterized with bipolar electrocautery.

### 5:40 Visual Appreciation of Disc.

A full-length view from top to bottom of the disc and the medially displaced spinal cord can be seen here. The epidural fat is seen pulsating through this newfound space created by this approach. The cautery device can be used dually as a dissector to carefully palpate the dorsal and medial aspects of the disc herniation.

### 6:18 Inferior Disc.

We can now appreciate the inferior border of the T10 pedicle and the disc fragment along with its hard shell, some soft disc material in the center, and the severely compressed spinal cord. As most of this disc was calcified, a significant amount of drilling was completed to thin the outer layer closest to the spinal cord for easier removal at the later portion of the case. Straight and slightly up-angled graspers removed the thinned rim of disc closest to the spinal cord. Epidural bleeders ventral to the spinal cord were cauterized carefully, and attention was placed on the most cephalad portion of the disc herniation. Kerrison rongeurs and graspers removed the medial rim of the disc.

### 7:16 Severe Compression and Final Removal of Cephalad Fragments.

The severely compressed spinal cord can clearly be seen here, almost folded over, with the last remaining calcified disc apparent at approximately the 3-o’clock position on the screen. A dissector can be seen here mobilizing the free fragment. Graspers were again used to remove the final compressive components of the disc.

### 7:50 Fully Decompressed Spinal Cord.

After the removal of the final compressive disc components, we can appreciate the now decompressed spinal cord in its most natural morphology.

### 8:00 Conclusions.

The < 1-cm incision was closed with absorbable sutures and dressed with glue. The postoperative MRI revealed complete resection of the thoracic disc and the caudally migrated fragment. The patient was discharged home 83 minutes after her surgery without the need for narcotic medications. Conclusively, we should also note that although these novel techniques can result in favorable outcomes, complex cases such as migrated large calcified thoracic discs can be challenging.^3–6^ The importance of training in full-endoscopic procedures should be emphasized, and these cases are likely best reserved for surgeons with sufficient clinical experience and volume in full-endoscopic spine procedures.^7^

## Disclosures

Dr. Konakondla reports being an educational consultant for Stryker and Joimax. Dr. Slotkin reports personal fees from Stryker and Medtronic, outside the submitted work.

## Author Contributions

Primary surgeon: Konakondla. Editing and drafting the video and abstract: Konakondla, Sofoluke. Critically revising the work: Konakondla, Barber, Slotkin. Reviewed submitted version of the work: all authors. Approved the final version of the work on behalf of all authors: Konakondla. Supervision: Konakondla, Slotkin. Provided clinical 3D models for surgery: Rimini.
